# Effects of pharmacological agents for neurogenic oropharyngeal dysphagia: A systematic review and meta‐analysis

**DOI:** 10.1111/nmo.14220

**Published:** 2021-08-01

**Authors:** Ivy Cheng, Ayodele Sasegbon, Shaheen Hamdy

**Affiliations:** ^1^ Centre for Gastrointestinal Sciences Division of Diabetes, Endocrinology and Gastroenterology School of Medical Sciences Faculty of Biology, Medicine and Health University of Manchester Manchester M6 8HD UK

**Keywords:** drugs, dysphagia, meta‐analysis, pharmacotherapy, systematic review, treatment

## Abstract

**Background:**

This systematic review and meta‐analysis aimed to evaluate the effects of pharmacological agents for neurogenic oropharyngeal dysphagia based on evidence from randomized controlled trials (RCTs).

**Methods:**

Electronic databases were systematically searched between January 1970 and March 2021. Two reviewers independently extracted and synthesized the data. The outcome measure was changed in (any) relevant clinical swallowing‐related characteristics.

**Key results:**

Data from 2186 dysphagic patients were collected from 14 RCT studies across a range of pharmacotherapies. The pooled effect size of transient receptor potential (TRP) channel agonists was large compared to placebo interventions (SMD[95%CI] =1.27[0.74,1.80], *p* < 0.001; *I* ^2^ = 79%). Data were limited for other pharmacological agents and the overall pooled effect size of these agents was non‐significant (SMD [95% CI] =0.25 [−0.24, 0.73]; *p* = 0.31; *I* ^2^ = 85%). When analyzed separately, large effect sizes were observed with Nifedipine (SMD[95%CI] =1.13[0.09,2.18]; *p* = 0.03) and Metoclopramide (SMD[95%CI] =1.68[1.08,2.27]; *p* < 0.001). By contrast, the effects of angiotensin‐converting enzyme (ACE) inhibitors (SMD[95%CI] = −0.67[−2.32,0.99]; *p* = 0.43; *I* ^2^ = 61%), Physostigmine (SMD[95%CI] = −0.05[−1.03,0.93]; *p* = 0.92) and Glyceryl Trinitrate (GTN) (SMD [95% CI] = −0.01 [−0.11, 0.08]; *p* = 0.78) were non‐significant. Within stroke patients, subgroup analysis showed that TRP channel agonists had a moderate pooled effect size (SMD[95%CI] =0.74[0.10,1.39]; *p* = 0.02; *I* ^2^ = 82%) whereas the effects of other agents were non‐significant (SMD[95%CI] =0.40[−0.04,0.84]; *p *= 0.07; *I* ^2^ = 87%).

**Conclusions & Inferences:**

Our results showed that TRP channel agonists, Nifedipine and Metoclopromide may be beneficial for neurogenic dysphagic patients. Large scale, multicenter clinical trials are warranted to fully explore their therapeutic effects on swallowing.

## Graphical abstract

Our systematic review and meta‐analysis found that transient receptor potential (TRP) channel agonists, Nifedipine and Metoclopromide may be beneficial for neurogenic dysphagic patients. Large scale, multicenter clinical trials are warranted to fully explore their therapeutic effects on swallowing.

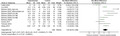

**Key Points**

- Transient receptor potential (TRP) agonists showed benefit in neurogenic dysphagia. Nifedipine and Metoclopramide may have therapeutic potential, but data are limited.
- Dysphagic stroke appeared to benefit more from TRP agonists than other pharmacological agents.
- Larger randomised controlled trials on pharmacotherapy for neurogenic dysphagia are warranted.

## 1 INTRODUCTION

Dysphagia is a symptom referring to difficulties in the passage of food or liquid from the mouth, through pharynx and esophagus, to the stomach.^1^ It can be anatomically classified into oropharyngeal dysphagia and esophageal dysphagia. Dysphagia affects approximately 56 million people worldwide^2^ and is prevalent among patients with stroke (8%–80%), Parkinson's disease (11%–81%) and traumatic brain injury (27%–30%), as well as community dwelling elderly people (11%–34%).^3^, ^4^, ^5^ Malnutrition, dehydration, aspiration pneumonia, prolonged hospital stay, mealtime anxiety and increased mortality are common physical and psychosocial consequences of dysphagia.^6^, ^7^, ^8^, ^9^ Moreover, the cost of healthcare resources is likely to be substantial for patients and to society in general due to their complex nature.^10^, ^11^ Dysphagia treatments are generally focused on improving safety and efficiency of swallowing. They can be compensatory, such as modifications of diet consistency or feeding posture, or rehabilitative, such as strength or skill training exercises for swallowing musculature.^12^ Rehabilitative interventions also include acupuncture, peripheral sensory stimulation through thermal, tactile or electrical (neuromuscular or pharyngeal) stimulation or non‐invasive brain stimulation including repetitive transcranial magnetic stimulation (rTMS) or transcranial electrical stimulation (TES).^12^

Of importance to this field, pharmacological agents are a potential management option for dysphagia and yet they have received relatively little attention compared to other treatments. These agents either stimulate swallowing‐related neural pathways in the peripheral or central nervous systems or directly modifying muscular function.^13^ To date, the drug classes that have been studied in the area of swallowing and oropharyngeal dysphagia include transient receptor potential vanilloid 1 (TRPV1) agonists,^14^, ^15^, ^16^, ^17^, ^18^, ^19^, ^20^ transient receptor potential ankyrin 1 (TRPA1) agonists,^21^ transient receptor potential melastatin 8 (TRPM8) agonists,^22^ levodopa,^23^, ^24^, ^25^ other dopaminergic agents,^26^ calcium blocking agents,^27^, ^28^ dopamine D2 receptor antagonists,^29^ angiotensin‐converting enzyme (ACE) inhibitors,^30^ beta blockers,^31^ nitric oxide donors^32^ and acetylcholinesterase inhibitors.^33^

Studies have suggested that these drugs may improve the swallowing reflex or reduce incidence of aspiration pneumonia in dysphagic patients. However, the underlying therapeutic mechanisms of action of these drugs are poorly understood. One mechanism is stimulation of afferent neural pathways for swallowing, for example receptors (TRPV1, TRPA1 and TRPM8) located in the oropharynx,^34^ through TRP channel agonists.^14^, ^15^, ^16^, ^17^, ^18^, ^19^, ^20^, ^21^, ^22^ Another mechanism involves increasing the level of or decreasing degradation of substance P, which is a neuropeptide known to enhance the swallow reflex,^35^ through capsaicin, ACE inhibitors or beta blockers.^17^, ^30^, ^31^, ^36^ Levodopa and dopaminergic agents may improve swallowing through improving dopamine metabolism.^23^, ^24^, ^25^, ^26^ Some studies have also suggested that treating coexisting esophageal dysphagia or facilitating stroke recovery may result in overall improvement in swallowing function.^27^, ^28^, ^29^, ^32^ Finally, physostigmine may improve swallowing in patients with progressive supranuclear palsy through cholinergic stimulation actions, but no significant effect has been reported.^33^

Given the scarce knowledge of the therapeutic potentials of pharmacological agents, this systematic review and meta‐analysis aimed to analyze their group effects on swallowing‐related outcomes in neurogenic oropharyngeal dysphagia from existing randomized controlled trials (RCTs). Further subgroup analysis was carried out to analyze the effects of these agents on stroke patients as strokes are the commonest cause of neurogenic dysphagia. The findings from our meta‐analysis should provide insights into the future research directions on pharmacological interventions for dysphagia.

## 2 MATERIALS AND METHODS

This review of data followed the Preferred Reporting Items for Systematic Reviews and Meta‐Analyses (PRISMA) guidelines. Two reviewers performed the search for studies, data extraction and risk of bias assessment independently. Data synthesis was carried out by one reviewer and verified by a second reviewer. Disagreements were resolved by consensus among all authors.

### 2.1 Study identification and search method

We searched the following electronic databases from January 1970 to March 2021: PubMed, EMBASE (via Ovid), CINAHL plus and Cochrane Library. Citations from identified papers were tracked and systematic reviews were searched manually for relevant references. The terms used for searches included dysphagia, swallowing disorders, deglutition disorders, swallowing, deglutition, pharmaceutical, drug, agent, medication and pharmacotherapy.

### 2.2 Eligibility criteria

We included only RCTs that compared pharmacological intervention with placebo intervention for neurogenic oropharyngeal dysphagia. Case studies, open‐label studies, animal studies, observational studies, quasi‐experimental studies, retrospective studies and studies that did not include original data were excluded. Non‐English studies were also excluded.

#### 2.2.1 Participants

Studies with adult patients with neurogenic oropharyngeal dysphagia (ie, dysphagia resulted from damage or deterioration of the central or peripheral nervous system) as determined clinically or through validated self‐report questionnaires regardless of the time of onset were included. Studies with healthy volunteers, patients without dysphagia or patients with esophageal dysphagia only were not considered. For studies that included both patients with and without dysphagia, only data from patients who were considered dysphagic, based on modified diet or at an elevated risk of aspiration pneumonia were extracted and analyzed.

#### 2.2.2 Interventions

We included studies that compared pharmaceutical interventions with placebo intervention. Trials with multiple interventions (eg, co‐administration of pharmacological agents and other swallowing therapies) were eligible if the study groups only differed by the use of the target pharmaceutical intervention of interest.

#### 2.2.3 Outcomes

Study outcomes related to swallowing, which included swallowing physiology measurement, clinical swallowing function ratings, functional dysphagia symptom scales or health outcomes related to swallowing functions, for example incidence of aspiration pneumonia, were included for comparisons. Studies that used non‐validated subjective rating of swallowing ability as an outcome measure were excluded.

### 2.3 Data extraction

The data extracted included: demographic information of participants (age and patient characteristics), intervention protocol (drug strength and dosage regimen), outcomes (mean [standard deviation; SD] or mean [95% confidence interval; 95% CI]) and sample sizes. For studies with multiple outcome measures, the most relevant primary swallowing‐related outcome was used. If data were not provided, we attempted to contact the corresponding authors. If data were presented in figures and raw data was not obtainable from the authors, an online plot digitalizer program (WebPlotDigitizer 4.3; https://apps.automeris.io/wpd/; USA) was used to extract graphic data. If data were not obtainable for quantification and analysis despite these attempts, the study was excluded from the review.

### 2.4 Risk of bias assessment

Seven domains of risk of bias of RCTs were evaluated using the Cochrane Collaboration's tool for assessing risk of bias.^37^ These include random sequence generation, allocation concealment, blinding of participants and personnel, blinding of outcome assessment, incomplete data, selective reporting and other sources of bias. Two reviewers rated the risk of bias of the included studies independently. Any disagreement on the judgements was discussed and resolved among all authors.

### 2.5 Statistical analysis

All statistical analyzes were performed by Review Manager 5.4 software program (RevMan; Cochrane Collaboration, Oxford, UK). The treatment effects were determined by comparing the treatment outcomes against that of the comparators. Studies with multiple interventions groups were analyzed separately for each experimental‐control comparison. Data extracted for treatment effect calculation included group sizes, group mean differences and pooled SDs. Pooled SD was calculated using the following formula ^38^:

$$SD_{\text{pooled}}=\sqrt{\frac{\left(n_{\text{pre}}‐1\right)SD_{\text{pre}}^{2}+\left(n_{\text{post}}‐1\right)SD_{\text{post}}^{2}}{n_{\text{pre}}+n_{\text{post}}‐2}}$$

Confidence intervals (CIs) were converted to SDs as suggested in the Cochrane Handbook.^37^ For outcome measures that increase with disease severity, the mean values were multiplied by −1. Treatment effects for continuous outcomes were analyzed as standardized mean difference (SMD) with 95% CI. A weighted average of SMD across studies was computed using a random effects model analysis. The significance level was set at *p* < 0.05 and the effect sizes were presented as SMD [95% CI]. For the interpretation of effect sizes, SMD of 0.2 represented a small effect, 0.5 a moderate effect, and 0.8 a large effect.^38^ Heterogeneity was assessed with Cochrane's *Q* statistic and *I* ^2^ test in which heterogeneity was considered substantial with *p* < 0.05 and *I* ^2^ higher than 50%.

## 3 RESULTS

**FIGURE 1:**
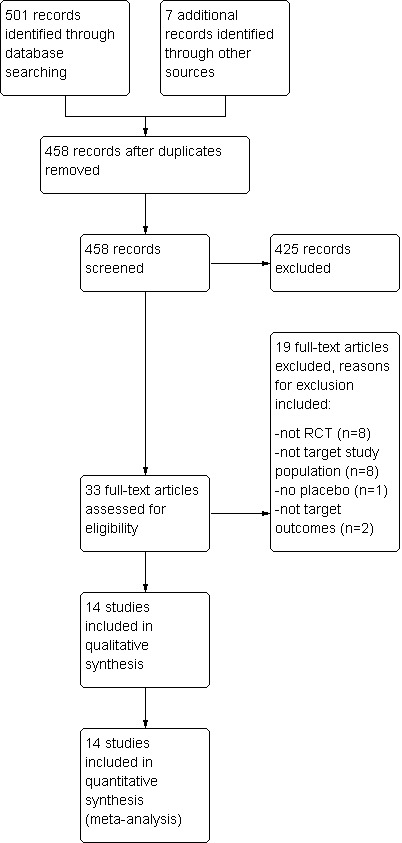
Flow diagram for study identification and inclusion.

Figure 1 shows the flow diagram of study identification. A total of 501 studies were identified from 4 electronic databases and 7 from other sources, of which 458 studies were considered potentially relevant. Fifty duplicated studies were removed and 425 studies were excluded by screening the titles and abstracts. Thirty‐three studies went through full‐text assessment of eligibility and we excluded 19 studies for reasons including not a randomized controlled trial, non‐relevant study population, no placebo intervention and no target outcomes of relevance. Fourteen studies met the inclusion criteria and were included in systematic review and meta‐analysis.

### 3.1 Study characteristics

The included studies were all published between 1998 and 2020. The total number of patients included in this meta‐analysis was 2186. Eight studies investigated the treatment effects of TRP channel agonists (TRPV1, TRPA1 and TRPM8 agonists) with 327 patients. One study investigated each of the following pharmacological agents: Lisinopril (ACE inhibitor; n = 71), Imidapril hydrochloride (ACE inhibitor; n = 54), Nifedipine (calcium blocking agent; n = 14), Metoclopramide (dopamine D2 receptor antagonist; n = 60), Physostigmine (acetylcholinesterase inhibitor; n = 8) and Glyceryl Trinitrate (GTN; nitric oxide donor; n = 1652). The mean age (SD) across all patients was 70.8 (12.2) years. Patients included in these studies had oropharyngeal dysphagia associated with stroke, aging, Parkinson's disease or progressive supranuclear palsy. Table 1 summarizes the characteristics of all included studies.

**TABLE 1:** Characteristics of included studies.

Study	Pharmacological agent	Drug strength; Dosage regimen	Comparison	Patient characteristics	Sample size	Age (years) Mean (SD)	Follow‐up schedule	Swallowing‐related outcome
TRPV1, TRPA1 and TRPV8 agonists								
44	Capsaicin	10μM (oral); Single dose	Active vs placebo (cross‐over)	Stroke	12	74.3 (7.8)	Immediately post	PAS
14	Capsaicin	150μM (oral); 2×/day for 21 days	Active vs placebo	Stroke	46/46	58.7 (7.4)	Immediately post	SSA
16	Capsaicin	0.025% ointment on external auditory canal; Single dose	Active vs placebo	Elderly with stroke or PD	10/10	80.3 (7.7)	5, 30 and 60 minutes post	ESS
18	Capsaicin	10 ml of 10 μM (oral); Single dose 10 ml of 10 μM (oral); 10 days	Active vs placebo Active vs placebo	Elderly with OD associated with aging	7/7 7/7	83.5 (6.3) 79.4 (5.3)	5 days post	PAS
19	Capsaicin	(Thermal tactile stimulation +nectar bolus) 150 μM/L; 3x/day for 21 days	Capsaicin vs distilled water	Stroke	30/30	65.0 (12.0)	Immediately post	SSA
20	Capsaicin	1 to 0.1 μM (oral); 3x/day for 28 days	Active vs placebo	Elderly	16/18	81.9 (1.4)	Immediately post	LTSR
15	Black pepper oil	(Concentration not specified) Nasal inhalation for 1 minute; Single dose	Black pepper oil vs distilled water	Stroke	34/33	85.0 (5.5)	Immediately post	LTSR
22	Menthol	10mM, 1mM and 100μM menthol (oral); Single dose	Distilled water vs various menthol concentrations vs cold distilled water (cross‐over)	Elderly	14	88 (3)	Immediately post	LTSR
Angiotensin‐converting enzyme (ACE) inhibitors								
30	Lisinopril	2.5 mg (oral); 1x/day for 26 days	Active vs placebo	Stroke patients with tube‐feeding	33/38	83.9 (6.2)	Week 12 post	RBHOS
54	Imidapril hydrochloride	1.25 mg, 0.625 mg, 0.5 mg, 0.25 mg (oral); Single dose	Active vs placebo	Stroke patients with silent aspiration	42/12	Not reported	Immediately post	Number of patients with silent aspiration
Calcium blocking agents								
27	Nifedipine	30 mg (oral); 1x/day for 28 days	Active vs placebo	Stroke	6/8	77.0 (6.3)	Immediately post	Pharyngeal transit time
Dopamine D2 receptor antagonists								
29	Metoclopramide	10 mg (oral); 3x/day for 21 days	Active vs placebo	Stroke patients with NGT and without pneumonia	30/30	78.1 (8.8)	Immediately post	Number of episodes of pneumonia
Acetylcholinesterase Inhibitors								
33	Physostigmine	1.25 ± 0.2 mg (optimal dose for each patient); 6x/day for 10 days	Active vs placebo (cross‐over)	PSP	8	64 (2.4)	3^rd^ or 4^th^ day	Swallow duration
Nitric oxide donors								
32	Glyceryl trinitrate (GTN)	5 mg (dermal patch); 1x/day for 7 days	Active vs placebo	Stroke	841/811	70 (12)	Immediately post	Feeding route

Abbreviations: ESS, Endoscopic Swallowing Score; LTSR, latent time of swallowing reflex; NGT, nasogastric tube; OD, oropharyngeal dysphagia; PAS, Penetration Aspiration Scale; PD, Parkinson's disease; PSP, progressive supranuclear palsy; RBHOS, Royal Brisbane Hospital Outcome Measure for Swallowing; SSA, Standardized Swallowing Assessment; TRPA1, transient receptor potential ankyrin 1; TRPM8, transient receptor potential melastatin 8; TRPV1, transient receptor potential vanilloid 1.

### 3.2 Risk of bias assessment

**FIGURE 2:**
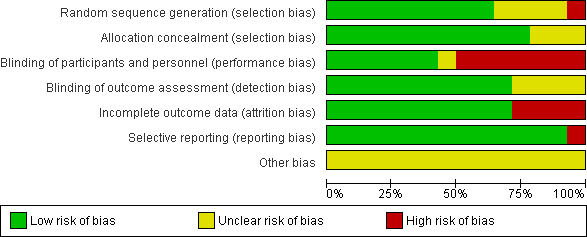
Risk of bias graph for all included studies.

**FIGURE 3:**
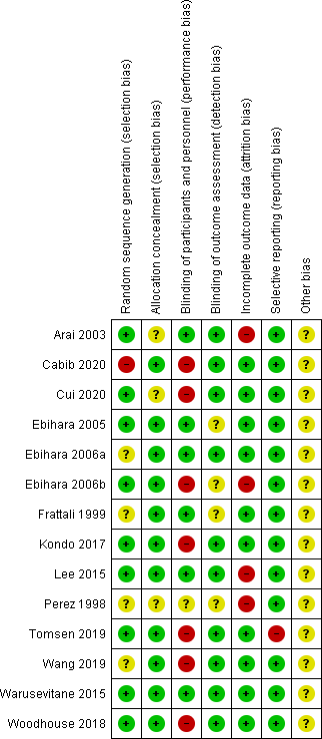
Risk of bias summary for individual studies.

The risk of bias assessment result is presented in Figures 2 and 3. Most studies had a low risk of selection and detection bias. Approximately half of the included studies had a high risk of performance bias due to the lack of blinding of personnel or participants. Attribution bias was high in 25% of the studies because of dropouts or deaths during the studies. Reporting bias was low for all but one study^18^ which did not report the outcomes of their control group in one of their sub‐studies. There was insufficient information to determine other risks so these could not be further quantified.

### 3.3 Outcome measures

The outcome measures used varied across studies. The most used outcome measures were clinical evaluation tools of swallowing functions, including Standardized Swallowing Assessment (SSA)^39^ and Royal Brisbane Hospital Outcome Measure for Swallowing (RBHOS),^40^ as well as dysphagia severity and swallowing safety evaluated based on instrumental evaluation, which included endoscopic swallowing scoring^41^ and Penetration Aspiration Scale (PAS).^42^ Five studies used timing of swallowing events, including pharyngeal transit time, swallow duration and the time between onset of bolus entering the pharynx and triggering of swallowing reflex (latent time of swallowing response; LTSR). The number of episodes of aspiration pneumonia and number of patients with silent aspiration were used in 2 studies. Finally, one study used feeding route, which was quantified by a scale comprising 7 levels including, 1: normal diet; 2: soft diet; 3: nasogastric tube; 4: percutaneous endoscopic gastrostomy tube; 5: intravenous or subcutaneous fluids; 6: no feeding/fluids and 7: death^32^, ^43^ as a clinical outcome measure.

### 3.4 Adverse events

Regarding serious adverse events, one study reported significantly higher mortality in the intervention (Lisinopril) group, which led to the premature termination of the study.^30^ Worsening of heart failure, flushing and giddiness were reported in the study with Nifedipine,^27^ although the relationships between these events and Nifedipine were not discussed by the authors. The GTN study reported that patients in the intervention group were more likely to have headache or clinical hypotension than the control group.^32^ No serious adverse events were reported with other pharmacological agents.

### 3.5 Dosage

The daily dosage ranged from once to six times whereas the overall course of intervention ranged from one to 28 days.

### 3.6 Meta‐analysis

#### 3.6.1 *Effects* *of pharmacological agents compared to placebo interventions*

**FIGURE 4:**
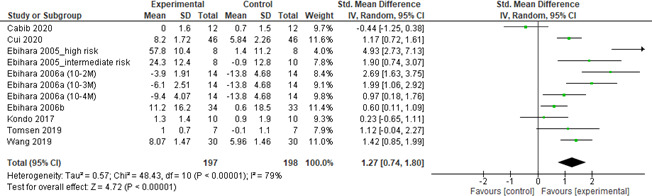
Forest plot showing pooled effects of transient receptor potential channel (transient receptor potential vanilloid 1 [TRPV1], transient receptor potential ankyrin 1 [TRPA1] and transient receptor potential melastatin 8 [TRPM8]) agonists compared to placebo interventions in patients with neurogenic dysphagia associated with stroke, aging, Parkinson's disease or progressive supranuclear palsy.

Among all drug classes, TRP channel agonists were studied most extensively with 8 RCTs. Therefore, a pooled effect size was computed for these agents. The results showed that TRPV1, TRPA1 and TRPM8 agonists yielded a large effect size with substantial heterogeneity (SMD [95% CI] =1.27 [0.74, 1.80], *p* < 0.001; *I* ^2^ = 79%) when compared to placebo intervention (Figure 4). Sensitivity analysis was thus carried out. The heterogeneity was slightly reduced (*I* ^2^ = 73%) when the study by Ebihara et al.,^22^ which employed a cross‐over design and was the only study that did not use TRPV1 agonist, and the study by Cabib et al.,^44^ which employed a cross‐over design and had high risk of randomization bias were excluded. The resulting pooled effect size remained large after adjustment (SMD [95%CI] =1.24 [0.85, 1.99]; *p* < 0.001).

**FIGURE 5:**
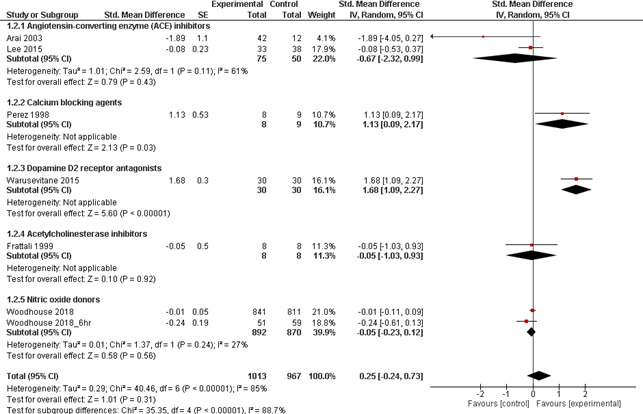
Forest plot showing pooled effects of other pharmacological agents, including angiotensin‐converting enzyme (ACE) inhibitors, calcium blocking agents, dopamine D2 receptor antagonists, acetylcholinesterase inhibitors and nitric oxide donors, compared to placebo interventions in patients with neurogenic dysphagia associated with stroke, aging, Parkinson's disease or progressive supranuclear palsy. Note that only one RCT was evaluable for the majority of drug classes except ACE inhibitors. For nitric oxide donors, *Woodhouse 2018_6hr* represented data from a subgroup of patients who received treatment within 6 hours of stroke onset as reported in the study by Woodhouse et al^32^

For the other pharmacological agents (Figure 5), only single or dual studies were evaluable, making interpretation less meaningful. Overall, the pooled effect size for these agents was non‐significant (SMD [95% CI] =0.25 [−0.24, 0.73]; *p* = 0.31; *I* ^2^ = 85%). When the effects of each drug class were analyzed separately, large and significant effect sizes were observed for Nifedipine (SMD [95% CI] =1.13 [0.09, 2.18]; *p* = 0.03) and Metoclopramide (SMD [95% CI] =1.68 [1.08, 2.27]; *p* < 0.001). By contrast, the pooled effect size of ACE inhibitors (Lisinopril and Imidapril hydrochloride) was non‐significant and negatively associated with beneficial swallowing outcome (SMD [95% CI] = −0.67 [−2.32, 0.99]; *p* = 0.43; *I* ^2^ = 61%). Similarly, the effect of Physostigmine (SMD [95% CI] = −0.05 [−1.03, 0.93]; *p* = 0.92) was non‐significant. Moreover, the effect sizes of GTN were non‐significant regardless of whether it was administered within 6 hours (SMD [95% CI] = −0.24 [−0.61, 0.14]; *p* = 0.22) or within 48 hours (SMD [95% CI] = −0.01 [−0.11, 0.08]; *p* = 0.78).

#### 3.6.2 *Effects* *of pharmacological agents on post*‐*stroke dysphagia*

**FIGURE 6:**
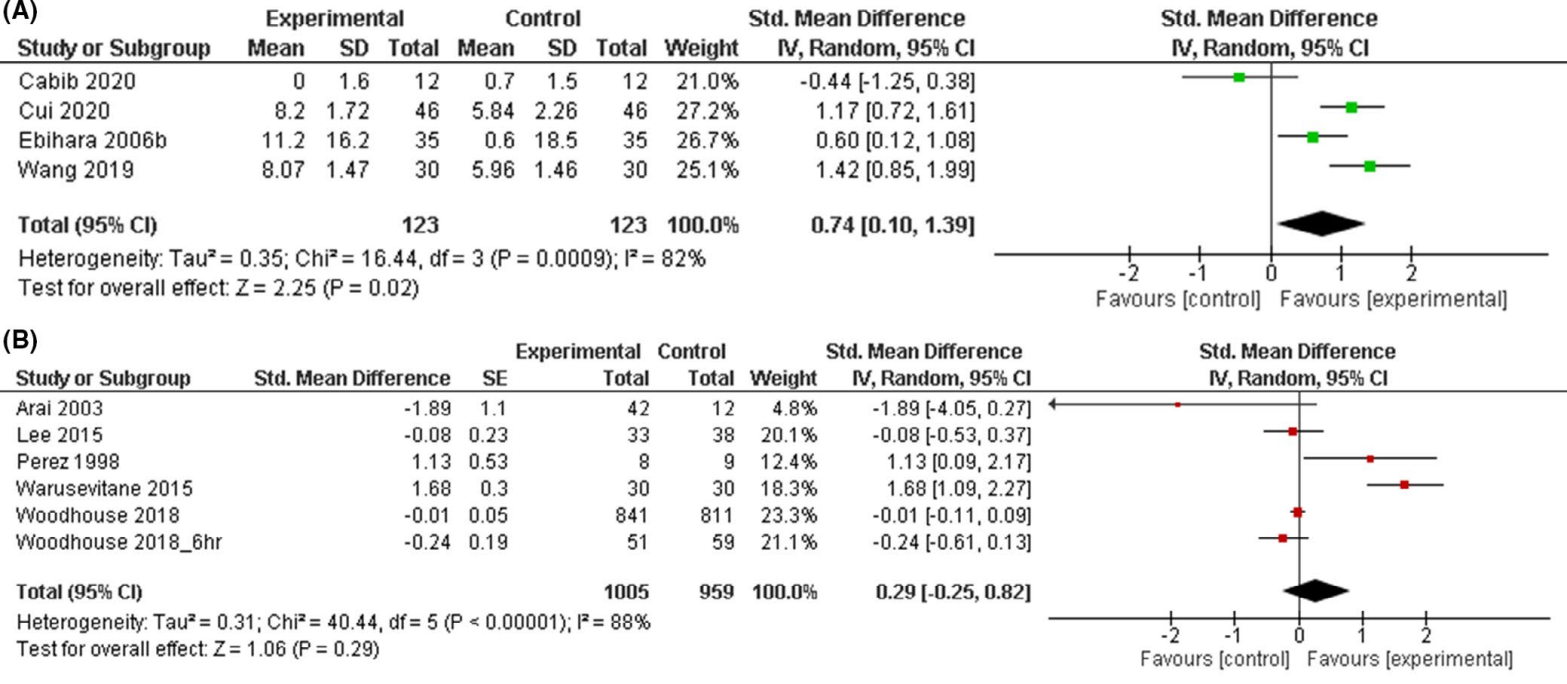
Forest plot showing pooled effects of (A) transient receptor potential channel (transient receptor potential vanilloid 1 [TRPV1], transient receptor potential ankyrin 1 [TRPA1] and transient receptor potential melastatin 8 [TRPM8]) agonists and (B) other agents compared to placebo interventions in stroke patients with dysphagia.

Given that stroke was the most studied disease group among all included studies (67%), a further analysis was carried out (Figure 6). TRP channel agonists showed a moderate pooled effect size with substantial heterogeneity (SMD [95% CI] =0.74 [0.10, 1.39]; *p* = 0.02; *I* ^2^ = 82%). The effects of other agents were analyzed as a group because only one RCT was available for most drug classes. The pooled effect size was non‐significant (SMD [95% CI] =0.29 [−0.25, 0.82]; *p *= 0.29; *I* ^2^ = 88%).

## 4 DISCUSSION

This systematic review and meta‐analysis evaluated the effects of pharmacological agents on swallowing‐related outcomes in (neurogenic) dysphagic patients. Among all drug classes, TRP channel agonists, predominantly capsaicin (TRPV1 agonist), were most extensively studied. We found that overall, TRPV1, TRPA1 and TRPM8 agonists are superior to placebo interventions with large effect sizes. The positive effects included reduced latency of swallowing response and dysphagia severity. By contrast, there are limited number of RCTs for other pharmacological agents such that their effectiveness remains questionable. Indeed, the pooled effect size of these agents was non‐significant. When each drug class was analyzed separately, calcium blocking agents (Nifedipine) and dopamine D2 receptor antagonists (Metoclopramide) showed large effect sizes. By comparison, ACE inhibitors (Lisinopril and Imidapril hydrochloride), acetylcholinesterase inhibitors (Physostigmine) and nitric oxide donors (GTN) showed no effects. Subgroup analysis on stroke patients showed that the pooled effect size of TRPV channel agonists was moderate whereas the effects of other agents were non‐significant. Adverse effects including increased mortality, worsening of heart failure, flushing, giddiness, headache and clinical hypotension were reported in studies with Lisinopril, Nifedipine and GTN. Our findings provided insights into the role and clinical value of pharmacological interventions for dysphagia which merits further discussion.

The functional changes following TRP channel agonists treatments may be a result of neuroplastic changes induced in the cortex by peripheral sensory stimulation of the corresponding receptors in the oropharynx. Sensory inputs are vital for triggering of swallowing as well as modulating motor swallowing response.^45^, ^46^ The oral and pharyngeal areas contain receptors that provide central nervous system information about texture, temperature, taste and dynamics of a food bolus as it passes along the swallowing tract.^46^, ^47^ TRPV1, TRPA1 and TRPM8 are examples of afferent receptors innervated by cranial nerves (trigeminal; CN V, glossopharyngeal; CN IX, vagus; CN X).^34^ They are sensitive to a range of temperatures and chemicals. TRPV1 can be activated by “hot” pepper (capsaicin) or heat (> 43℃) whereas TRPA1 and TRPM8 can be activated by cold stimuli.^48^, ^49^ TRPA1 responds to pungent stimuli such as wasabi or mustard and (unpleasant) low temperature (< 17°C); associated with burning pain sensation in extreme cold.^50^, ^51^ TRPM8 responds to milder stimuli such as menthol and temperature (25–28°C).^50^, ^51^ These receptors belong to the sub‐families of TRP channels. TRPs are cationic channels expressed at the plasma membrane which when activated, allow Ca^2+^ ions to enter and depolarize sensory neurons.^49^ This leads to triggering of sensory impulses, which are then transmitted to the nucleus tractus solitarius (NTS) of the medulla and the sensorimotor cortex through interneuronal connections.^45^, ^46^

Preliminary neurophysiological evidence appears to support the hypothesis that functional changes induced by TRP agonists are centrally mediated. Using electroencephalography (EEG), Tomsen et al.^18^ demonstrated that oral capsaicin increased cortical event‐related potentials in cingulate gyrus and medial frontal gyrus during swallowing, indicating an improved conduction and integration of sensory information into the cortex. Such neurophysiological changes were associated with improved swallowing responses in elderly patients with oropharyngeal dysphagia. Moreover, Cabib et al.^44^ reported enhancement in excitability of the motor cortex, albeit with no functional improvements, after oral capsaicin treatment in stroke patients. The lack of functional changes may be due to the low dosage (10μM; single dose) used for chronic (> 3 months) post‐stroke dysphagia. By comparison, a recent study with healthy volunteers found that despite inducing changes in swallowing biomechanics, oral capsaicin did not alter cortical activation patterns as revealed by magnetoencephalography (MEG).^52^ This negative finding may reflect the dual effects of capsaicin where the immediate pharyngeal swallow response may be improved by single dose administration, but long‐term neuromodulatory effects may only be induced through repetitive stimulation. Nonetheless, these findings provided valuable insights into the neurophysiological effects and dose‐dependency of TRP channel agonists. Large scale, multicenter clinical trials are warranted to further investigate the optimal dose for sustained neurophysiological and functional improvements.

Apart from sending sensory impulses to the central nervous system, TRPV1 agonists may modulate swallowing through releasing substance P, which is a neuropeptide that enhances cough reflex.^53^ Studies have found that reduced levels of substance P are associated with an increased risk of aspiration pneumonia in elderly patients,^35^ stroke patients^54^, ^55^ and patients with Parkinson's disease.^56^ Given that an increase in serum substance P level after capsaicin treatment has been reported in some RCTs,^14^, ^15^ it is possible that this neuropeptide may play a role in the observed improvements in swallowing function. The mechanisms of TRPV1 agonists on the release of substance P and the relationship between substance P and swallowing function are not fully understood. In healthy volunteers, Suntrup‐Krueger et al.^52^ found that the effects on salivary substance P level are dose dependent, where an increase was only detected with high dose (50μM) but not low dose (10μM) oral capsaicin. In elderly patients with dysphagia, a recent RCT found that increased levels of substance P is associated with improvement in swallowing efficiency following capsaicin treatment.^17^ Some studies have explored the relationship between substance P and the physiology of swallowing. Tomsen et al.^57^ found that elderly patients with oropharyngeal dysphagia showed impaired pharyngeal sensitivity compared to healthy volunteers and substance P level was negatively correlated with pharyngeal sensory threshold. Moreover, in acute stroke patients, low substance P level was associated with low frequency of spontaneous swallowing and increased incidence of pneumonia.^55^ These findings suggested that substance P level is closely related to swallowing performance and may be a potential marker for pharyngeal sensitivity or stroke‐related aspiration pneumonia.

Previous reports have suggested that ACE inhibitors may be beneficial to dysphagic patients by reducing degradation and inactivation of substance P.^58^ Arai et al.^54^ suggested that Imidapril hydrochloride may increase substance P and reduce the risk of silent aspiration in stroke patients, although the effect size was non‐significant in our meta‐analysis. In contrast, Lee et al.^30^ found that Lisinopril did not lower the risk of aspiration pneumonia nor improve swallowing functions. Concerningly, they also found a higher mortality rate in the active intervention group. Although these studies emphasized that the dose used was lower than the standard dose for hypertensive treatment, caution must be taken when administrating these drugs to avoid systemic effects on blood pressure, the cardiovascular and renal systems.

Our review found that there are very limited number of RCTs that investigate the effects of calcium blocking agents, dopamine D2 receptor antagonists, acetylcholinesterase inhibitors and nitric oxide donors on swallowing. Positive findings have been reported for the former two agents, but underlying mechanisms remain largely speculative.^27^, ^29^ Nifedipine is a calcium blocking agent that can be used to alleviate chest pain and rapidly lower blood pressure.^59^ Perez et al.^27^ postulated that Nifedipine may improve pharyngeal dysphagia through reducing coexisting esophageal spasm or through global enhancement on stroke recovery. Metoclopramide is a dopamine antagonist used to reduce nausea and vomiting.^60^ Warusevitane et al.^29^ suggested several possible mechanisms of Metoclopramide in reducing incidence of aspiration pneumonia. These include reduced regurgitation through increasing the tone of lower esophageal sphincter and accelerating gastric emptying in patients with nasogastric tube‐feeding. By contrast, negative findings were reported for Physostigmine (acetylcholinesterase inhibitor) in patients with progressive supranuclear palsy. The authors argued that the dose used was not sufficient to inhibit acetylcholinesterase activity in the central nervous system and cause any functional changes. Similarly, our meta‐analysis showed that the effects of GTN were non‐significant regardless of the time of administration. Such finding differed from the results reported by Woodhouse et al.^32^ in which they found that GTN could improve the route of feeding when it was given within 6 hours of stroke onset. This discrepancy may be explained by the exclusion of data from patients who were on normal diet (and presumably non‐dysphagic) in our analysis. Moreover, baseline data for dysphagic patients were not reported. GTN is a type of nitric oxide donor used to treat high blood pressure and heart failure and its early administration may have beneficial effects for stroke patients.^61^, ^62^ Woodhouse et al.^32^ proposed that the observed improvements on feeding route may be driven by a general facilitation of stroke recovery, rather than mechanisms specific for swallowing. Animal studies have shown that nitric oxide is important for initiation of swallowing and esophageal peristalsis.^63^ It is possible that nitric oxide donors may improve swallowing reflex through supplying nitric oxide exogenously, although this explanation remains speculative without pharmacodynamic evidence. Notwithstanding, some subtle treatment effects may have been missed in this rather more restrictive meta‐analysis. More RCTs are warranted for these pharmacological agents before they could be considered as potential interventions for dysphagia.

Our subgroup analysis showed that TRP channel agonists appeared to have larger positive effects for stroke patients compared to other pharmacological agents. Neuroplasticity, which is the reorganization of neural networks in response to damages or disruptions, plays an important role in stroke recovery. Specifically, improvement in swallowing function in unilateral stroke patients is driven by an increase in the cortical representation of the undamaged hemisphere.^64^ This might provide unique opportunities for pharmacological agents to alter outcome beyond peripheral effects. The positive neurophysiological effects reported with TRP channel agonists^18^, ^44^ may explain the larger effects compared to other agents. However, cautions must be taken when interpreting this result because of the high heterogeneity and a smaller number of RCTs for other agents than TRP channel agonists. Similarly, although the effects of these agonists appeared to be smaller in stroke patients than in patients with neurogenic dysphagia, the difference may be attributed to the smaller number of trials in stroke patients. Moreover, a mixed population of stroke patients with different severity and chronicity were studied in these RCTs. A recent meta‐analysis showed that the effects of neurostimulation treatments varied according to the chronicity of stroke.^65^ Therefore, it is plausible that the stroke characteristics may have influenced the responsiveness to TRP channel agonists, hence limiting their treatment efficacy in stroke patients.

The quality of studies included in our meta‐analysis was considered moderate due to the high risk of performance bias. Approximately half of the included studies did not have reliable blinding of participants or personnel. These were primarily studies with TRP channel agonists. While blinding is ideally done by delivering a placebo treatment that appears identical to the active treatment, it can be challenging for some compounds with strong, distinctive taste and smell such as TRP channel agonists. Moreover, placebo treatment may not be available from manufacturers^62^ such that a control condition that resembles the active treatment needs to be made from other materials, which may influence its validity. The use of an active control may minimize performance bias, but in some cases, single‐blinded designs may be unavoidable.

Our review is limited by the small number of studies. For some drug classes, only one RCT was eligible for analysis, making it difficult to draw any definitive conclusions regarding their efficacies. Given the small number, the risk of publication bias cannot be evaluated. Moreover, only English studies were included for analysis. Lastly, patient characteristics, outcome measures and intervention protocols of included studies were highly heterogeneous. Therefore, our results must be interpreted with some caution.

In conclusion, our systematic review found that TRPV1, TRPA1 and TRPM8 agonists have beneficial effects for patients with neurogenic oropharyngeal dysphagia when compared to placebo interventions. There are very limited number of RCTs for other pharmacological agents, including ACE inhibitors, calcium blocking agents, dopamine D2 receptor antagonists, acetylcholinesterase inhibitors and nitric oxide donors. Therefore, the treatment effects of these drugs remain uncertain. Major adverse effects reported include increased mortality, worsening of heart failure, flushing, giddiness, headache and hypotension in clinical trials with Lisinopril, Nifedipine and GTN. Overall, the level of evidence for pharmacological interventions for neurogenic oropharyngeal dysphagia remains low. Future large scale, multicenter clinical trials are warranted to fully explore the potential of these agents.

## CONFLICT OF INTEREST

All authors declare that there are no conflicts of interest.

## AUTHOR CONTRIBUTIONS

All authors contributed substantially to conception and design of the review, acquisition, analysis and interpretation of data, drafting the article and reviewing it critically for important intellectual content. All authors approved the final version of the article.
